# Correction for Khan et al., “Targeting the HIV-1 Spike and Coreceptor with Bi- and Trispecific Antibodies for Single-Component Broad Inhibition of Entry”

**DOI:** 10.1128/JVI.00391-19

**Published:** 2019-05-15

**Authors:** Salar N. Khan, Devin Sok, Karen Tran, Arlette Movsesyan, Viktoriya Dubrovskaya, Dennis R. Burton, Richard T. Wyatt

**Affiliations:** a Department of Immunology and Microbiology, The Scripps Research Institute, La Jolla, California, USA; b IAVI Neutralizing Antibody Center, The Scripps Research Institute, La Jolla, California, USA; c Center for HIV/AIDS Vaccine Immunology and Immunogen Discovery, The Scripps Research Institute, La Jolla, California, USA

## AUTHOR CORRECTION

Volume 92, no. 18, e00384-18, 2018, https://doi.org/10.1128/JVI.00384-18. The article was originally published on 29 August 2018 with a standard copyright line (“© 2018 American Society for Microbiology. All Rights Reserved.”). We elected to pay for open access for the article after publication, necessitating replacement of the original copyright line with “© 2018 Khan et al. This is an open-access article distributed under the terms of the Creative Commons Attribution 4.0 International license.” This change was made to the online version of the article on 29 March 2019.

